# Facilitators and barriers in the development and implementation of depression prevention and treatment policies in China: a qualitative study

**DOI:** 10.1186/s12889-023-15201-0

**Published:** 2023-02-07

**Authors:** Jinping Ma, Hai Zhou, Qinqin Fu, Guohua Lu

**Affiliations:** 1 School of Public health, Weifang Medical University, Weifang, China; 2 Weifang Mental Health Center, Weifang, China; 3 School of Psychology, Weifang Medical University, Weifang, China

**Keywords:** Depression control, Policy, Qualitative research

## Abstract

**Background:**

Depression is one of the leading causes of avoidable suffering and premature death worldwide, leading to the disease burden among mental disorders. Depression-related deaths can be prevented by developing and implementing good depression prevention and treatment policies. The goal of this study is to provide theoretical direction and useful references for examining the outstanding service work of depression prevention and treatment. It also aims to describe how depression prevention and treatment policies were developed and put into practice in China, along with the associated facilitators and barriers.

**Methods:**

We integrated two data sources using a case study approach: a document review of relevant policy documents, published articles and reports between 2004 and 2022 (N = 12 papers) and in-depth interviews (N = 41). Participants were drawn from pertinent sectors to managing depression: research and academia, relevant government departments, health care providers, people with depression and their families, and community organisations. Thematic analysis was used to analyse all data.

**Results:**

A comprehensive programme of work exploring specific services for depression prevention and treatment was developed in China in 2020. Facilitators of policy development and implementation include (1) political commitment and strong leadership, (2) coordination mechanisms, (3) stakeholder enthusiasm and commitment, (4) resources, and (5) the use of digital technologies. The main barriers leading to delays in policy development and implementation include (1) insufficient awareness and lack of depression literacy, (2) lack of resources and (3) stigma and social discrimination (4) lack of united action.

**Conclusion:**

Although the process of implementing a distinctive service programme for depression prevention and treatment in China has been long, the current policy is in line with current global efforts. Strategies to reduce Stigma and increase knowledge about depression are part of a national and international approach to reducing the burden of depression. Political commitment and the involvement of all stakeholders remain necessary. An adequate response to depression will require the involvement of society as a whole, with joint action to reduce the risk of exposure to adversity and enhance protective factors.

## Background

Depression is a major global public health problem with high prevalence, recurrence and mortality rates and is a significant contributor to the worldwide burden of disease [1]. According to a study published in 2019 by the Global Burden of Disease Study, depressive disorders account for the highest disease burden among mental illnesses. According to the World Health Organization (WHO) 2020, more than 264 million people worldwide suffer from depression, and approximately 850,000 deaths are depression-related each year. Studies have found that the lifetime prevalence of adult depressive disorder in China is 6.8% [2]. The analysis also showed that the prevalence of depression among Chinese women was 4.2%, significantly higher than that of men (3.0%); the age group 50–64 had the highest prevalence of depression at 4.1%; and the prevalence of depression among rural people was 3.7%, slightly higher than that of urban people (3.4%). The Report on the Development of National Mental Health in China shows that the detection rate of depression among adolescents in China is 24.6% [3].

Evidence has accumulated over decades that depression is one of the leading causes of avoidable suffering and premature death worldwide. However, not enough has been done to avoid and alleviate the suffering and disadvantages associated with depression, and few governments have acknowledged the impediments to social and economic development caused by depression. Studies have shown that people with depressive disorders have significant impairments in social functioning but have low utilisation of health services and rarely receive adequate treatment. Only 9.5% of people with depressive disorders in China were treated, and only 0.5% were adequately treated [2]. Although solid evidence of the effectiveness of some intervention strategies at multiple levels of promotion, prevention, treatment and support, a lack of awareness of mental health and depression and significant Stigma and discrimination continue to hinder public action [4]. Therefore, the government needs to develop plans to increase health resources’ availability, accessibility and acceptability to facilitate increased health service utilisation for people with depressive disorders.

The Party and the State attach great importance to the nation’s mental health and increasing importance to mental health work. On October 25 2016, the Central Committee of the Communist Party of China and the State Council issued and implemented the “Health China 2030” Planning Outline, proposing to increase the promotion of mental health science for the whole population, improve mental health literacy and strengthen interventions for common mental disorders such as depression and anxiety disorders. On September 11 2020, to implement the “Health China Action (2019–2030)” mental health promotion activities, increase the prevention and treatment of depression, and curb the rising trend of prevalence, the General Office of the National Health and Wellness Commission issued the Work Plan for Exploring Special Services for the Prevention and Treatment of Depression, requiring the organisation and implementation of a particular pilot for the prevention and treatment of depression. This study aims to describe the development and implementation of depression prevention and treatment policies in China, identify relevant barriers and facilitators, and provide theoretical guidance and practical references for exploring special services for depression prevention and treatment.

## Method

### Study design

In this paper, we use a case study design that facilitates the investigation of phenomena over time using various data sources to determine true quality [5]. The document review provided context and historical background [6]. Key informant interviews were conducted with stakeholders to obtain information about events and understand participants’ perceptions of policy development and implementation.

### Document reviews

Databases were searched to find studies on depression control policy documents in China. We searched PubMed and Google using different keyword combinations of search terms [depression control policies + China + barriers + facilitators]. We considered all documents from 2004 to 2022. Also, We searched the online websites of government agencies such as the Ministry of Health, Ministry of Education, Ministry of Propaganda and Ministry of Civil Affairs. We also conducted manual searches of the National Centre for Mental Health and Mental Hygiene Control, government libraries and government officials’ offices, targeting documents such as programmes, laws, strategic plans, guidelines and government directives. Data extraction forms were used to collect information on multisectoral methodological elements such as policies, year of publication, policy objectives, key players in the policy process, interventions, and stakeholder engagement. This data extraction search was conducted independently by two researchers. Results were compared, reconciled in areas of inconsistency, and integrated.

### Key informant interviews

The document review guides the selection of key informants. Key informants actively involved in the decision-making process were invited to participate if they were listed in the document or reasonably expected to be involved in the decision-making process based on the document review.

The initial research participants were purposefully selected to provide organisational representation and variation in decision-making roles. In our study, interviewees were purposefully selected by the researcher based on empirical principles from organizations or individuals involved in depression prevention and treatment programs in order to obtain a more in-depth and detailed interpretive understanding based on the objectives of the study. When interviewing the initial key informants, other key informants were identified using a snowball technique [7]. Every effort was made to contact and interview individuals involved in decision-making/implementation if they were no longer in their roles. A total of 41 key informants from different industries were interviewed. The development of the work program for the specialized services for depression prevention and treatment, the context and content of policy, participants in policy development, policy implementation, barriers and facilitators to policy development and performance, and suggestions or recommendations on how to improve the development and implementation process were among the information gathered in the Key Informant Interview Guide.

### Data collection

We conducted face-to-face interviews with key informants from public and private organisations working in sectors related to depression prevention and treatment in China, particularly about the development and implementation of depression prevention and treatment policies. Health, education, advocacy, media, and voluntary and community organisations were among these sectors. Data were collected between January and June of 2022. A semi-structured interview guide was used to conduct the interviews. Interviews have taken place in the offices of critical informants or their preferred private locations. Discussions took an average of 30–60 min to complete. All interviews were transcribed verbatim into a Word document.

### Data analysis

The data were analysed through thematic content analysis [8]. Walt and Gilson’s policy triangle framework focuses on four areas: content, context, process and the participants who play a crucial role in policy development [9]. Interview transcripts were entered into NVivo 10 software, which helps manage data ideas, queries, visualisation, and reporting [10]. Two researchers independently examined 10% of the coded transcripts (N = 4) and found 83% inter-coder reliability, which was sufficient [11].

## Ethical considerations

This study was approved by the Ethics Committee of Weifang Medical College. to participate in the survey, to understand its potential risks and benefits, confidentiality, and their right to withdraw from the study at any time without penalty. Participants gave verbal and electronic informed consent to participate in this study.

## Results

### Data sources

#### Document reviewed

We identified six national depression control policies, three published articles and one Report on depression control policies (see Table 1).

**Table 1:** List of documents analysed

Document Type	Document title	Author	Year
Policy Documents	Exploring the Work Programme of Specialized Services for Depression Prevention and Treatment	General Office of the National Health and Wellness Commission	2020
	Healthy China Action (2019–2030)	State Council of the Central Committee of the Communist Party of China	2019
	Outline of the “Health China 2030” Plan	State Council of the Central Committee of the Communist Party of China	2016
	The 13th Five-Year Plan for Health and Wellness	State Council of the Central Committee of the Communist Party of China	2016
	National Mental Health Work Plan (2015–2020)	State Council of the Central Committee of the Communist Party of China	2015
	Guidance on further strengthening mental health	State Council of the Central Committee of the Communist Party of China	2004
Journal articles	It’s time to take action against depression	China Science News	2022
	Explaining the programme of work to explore special services for depression prevention and treatment	Internal Medicine	2021
	There is an actual increase in the number of people suffering from depression.	China Youth Daily	2021
Reports	China National Mental Health Development Report (2019–2020)	Fu Xiaolan, Zhang Kan, Chen Xuefeng, Chen Zhiyan	2021

## Key informant interviews

Forty-one individuals from different sectors were interviewed (see Table 2). Most participants were from the health administration, although they came from other parts of the health system. Additional health system personnel, such as health department officials, mental health specialists and researchers, psychiatric hospital medical staff, general hospital medical staff, psychotherapists and counsellors, are examples. Some participants include vital populations (people with depression and their families, adolescents, the elderly, pregnant women, etc.), community organisations and voluntary organisations.

**Table 2:** Number of respondents interviewed by sector

Sector	Category	Number
Health	Health sector officials	2
	Mental Health Specialists and Researchers	3
	Medical staff in the psychiatric hospital	4
	Medical staff in the general hospital	4
	psychotherapists	2
	counsellor	2
Education	Education Department Officials	2
	Staff	1
publicity department	Publicity department officials	1
	Staff	1
Community	Community Residents	2
	Resident Council Members	2
	Social Worker	2
Volunteer Organisations	Volunteers	2
	Members of mutual-help group	2
Key populations	patients with depression and their families	4
	Youth	2
	Older people	2
	Maternity	1
Total		41

## Policy background on depression prevention and treatment

The findings of the document review and key informant interviews suggest that vital global initiatives and local epidemiological and political factors have combined to influence depression prevention and treatment policy.

**Global context** In a worldwide context, depression is a shared global condition that affects an estimated 3.8% of the population [12]. Approximately 280 million people in the world suffer from depression [12]. Although there are proven treatments for mental disorders, more than 75% of patients in low- and middle-income countries do not receive treatment [13].

To this end, the WHO Mental Health Action Plan 2013–2030 highlights the measures needed to provide appropriate interventions for people with mental disorders, including depression. Depression is a priority disorder addressed by the WHO Mental Health Gap Action Plan (mhGAP) [14]. The goal of the plan is to promote increased services for people with mental, neurological and substance abuse in countries through the provision of care by health workers who are not mental health specialists; in addition, which has developed a short manual of psychological interventions for depression that can be delivered to individuals and groups by non-professionals. In summary, the formulation and implementation of depression prevention and treatment policies in China have been influenced to some extent by relevant WHO policies.

**Local epidemiological background** Epidemiological factors are essential in implementing depression prevention and treatment programmes in China. The study found that the lifetime prevalence of the depressive disorder among Chinese adults was 6.8% [2]. The Report on the Development of National Mental Health in China, published by the Institute of Psychology, Chinese Academy of Sciences, shows that the detection rate of depression among adolescents in China is 24.6%, of which the detection rate of major depression is 7.4%. In contrast to the high prevalence rate, the identification and consultation rates for depression are extremely low, averaging only about 10%.

**Political background** The Party and the State attach great importance to the nation’s mental health. To promote the construction of a healthy China and improve people’s health, the Central Committee of the Communist Party of China and the State Council issued and implemented the “Health China 2030” Planning Outline on October 25 2016, proposing to increase the popularisation of mental health science for all people and improve mental health literacy. 2020 On September 11 2020, the National Health Office released the Work Plan for Exploring Special Services for the Prevention and Treatment of Depression, which includes the prevention and treatment of depression as a particular pilot project.

Mental health is an essential part of health. The Party and the government attach great importance to mental health services, pay attention to the nation’s mental health, and require localities to promote mental health promotion actions actively and gradually improve China’s mental health service system. -Health department official 1.

## Depression Prevention and Control Policy Content

The Work Programme for Exploring Special Services for Depression Prevention and Treatment is China’s central depression prevention and treatment policy. This programme covers topics such as strengthening knowledge and education, conducting screening and assessment, improving the capacity for early diagnosis and standardised treatment, increasing interventions for key populations, strengthening psychological hotline services and timely psychological interventions. Key elements in the depression control policies in China are summarized in Table 3.

**Table 3:** Elements of depression control policies in China

Policy /Year	Elements in the policies
Exploring the Work Programme of Specialized Services for Depression Prevention and Treatment	We will strengthen education on prevention and treatment, carry out screening and assessment, improve early diagnosis and standardised treatment, intensify interventions for key populations, strengthen psychological hotline services, and carry out timely psychological interventions.
National Mental Health Work Plan (2015–2020)	Explore models of prevention and treatment for common mental disorders that are suitable for the actual situation in the region, and encourage areas with conditions to provide follow-up services for depressed patients.
Healthy China Action (2019–2030)	To popularise the application of psychological counselling and treatment techniques in clinical diagnosis and treatment and to promote the application of special techniques and methods of psychological adjustment in Chinese medicine.
Outline of the “Health China 2030” Plan	Interventions for common mental disorders such as depression are strengthened, and early detection and timely intervention for psychological problems in key populations are increased.
The 13th Five-Year Plan for Health and Wellness	To gradually establish and improve the community rehabilitation service system for people with mental disorders and to carry out pilot early screening and intervention for common mental illnesses like depression.

## Policy process

### Agenda setting and policy development

According to the document review, the discussion on the prevention and treatment of depression in China began in 2013 with the explicit definition of depression as a mental illness in the Mental Health Law of the People’s Republic of China. On October 25 2016, the Central Committee of the Communist Party of China and the State Council issued and implemented the “Health China 2030” Planning Outline, proposing to increase the popularisation of mental health science for the entire population, strengthen interventions for common mental disorders such as depression and anxiety disorders, and improve people’s health. The General Office of the National Health and Wellness Commission issued the “Exploring Depression Prevention and Treatment Specialized Services Work Plan” on September 11, 2020, and designated depression prevention and treatment as a pilot featured project.

## Policy implementation status

Participant comments and document review indicated that some of the provisions of the Distinctive Service Work Programme for Depression Prevention and Control had been implemented, and related interventions have been implemented to some extent.

## Promoting depression literacy through knowledge about depression

Under the leadership of party committees and governments at all levels in the pilot areas, health and propaganda departments have strengthened their collaboration and used various propaganda means to publicise the scientific knowledge of depression widely.

## Building a grassroots psychological service platform

The pilot areas have established three-tier psychosocial service platforms at the district (county), street (town) and community (village) levels, following the work requirements of the Central Office of Comprehensive Governance.

## Conducting depression screening

Health care facilities use the PHQ-9 scale to screen for depression. All medical examination centres include assessment of emotional state in their medical examination programmes for use by medical examiners. Individual high schools and higher education institutions include depression screening in student health check-ups, establishing student mental health records, assessing students’ mental health status and giving focused attention to students with abnormal assessment results.

## Enhanced training to improve capacity for standardised treatment

Standardised and continuous depression prevention and treatment training and other related knowledge in medical and health institutions at all levels. Training for physicians in non-psychiatric hospitals is stepped up to improve their ability to recognise depression and make timely referrals.

## Enhanced psychological hotline service

Establish a 24-hour psychological support hotline that offers public service, relying on mental health medical institutions or professional forces like the 12,320 public health public service hotline and social and cognitive health service organizations.

## Establishing a psychological crisis intervention team for timely psychological intervention

Establish and improve a professional psychological crisis intervention team, including psychiatrists, psychotherapists, psychologists, and social workers. Organise psychological counselling and psychological intervention in the event of major infectious diseases, natural disasters and other emergencies to prevent and reduce the occurrence of extreme behaviour.

## Actors

The policy process was consultative and involved multiple government departments and other stakeholders. Interviews with informants revealed that actors from different health and government departments participated in the survey, including government organisations, health departments, advocacy departments, education departments, community-based organisations, civil society organisations and voluntary organisations. Table 4 summarises the roles played by the different institutions, sectors and stakeholders in the study of the policy implementation process.

**Table 4:** The role of actors in the implementation of depression prevention and treatment policies

sector/stakeholder	Role in policy development and implementation
Health	Strengthen education on prevention and treatment; carry out screening and assessment; improve early diagnosis and standardised treatment capacity; strengthen psychological hotline services, and carry out a timely psychological intervention.
publicity department	Strengthen collaboration with the health sector, adopt various publicity means, and use multiple channels such as film and media to publicise the scientific knowledge of depression widely.
Education	Depression screening is included in students’ medical examinations, setting up mental health education courses staffed by mental health teachers.
Civil Affairs	Strengthen collaboration with other departments to implement various tasks.
Public Security	Strengthen collaboration with other departments to implement various tasks.

## Facilitators of policy development and implementation for depression prevention and treatment

Several vital facilitators have been described in the development and implementation of depression prevention and treatment policies.

1. **Political commitment and strong leadership.** Participants pointed out that strong government leadership is the key to implementing policies for the prevention and treatment of depression. Local governments should incorporate mental health work into national economic and social development planning, include it in the government’s work agenda, propose mental health work goals and coordinate planning according to the actual situation in the region.
2. **Existence of coordination mechanisms.** The involvement of many stakeholders meant that a stakeholder coordination mechanism was necessary to guide the policy process. The coordination mechanism is therefore described as a facilitating factor in the implementation process. At the national level, a mechanism was established for united action by the Ministry of Education, the Health and Welfare Commission, the Central Committee of the Youth League and other departments to strengthen the division of labour.

Departments, units and groups such as health, civil affairs, public security, education, justice, the Disabled Persons’ Federation, the Communist Youth League, the Women’s Federation and the Committee on Ageing are required to take effective preventive and control measures in response to the increasingly prominent mental health problems, step up efforts within their respective areas of responsibility, and strengthen coordination and collaboration to create synergy.- Government officials 1.

(3) **Stakeholder enthusiasm and commitment.** Passion and commitment were reported to be necessary for policy development and implementation. These characteristics were mainly found in advocates or champions, who made the policy environment for depression prevention and treatment dynamic. Interviewees also indicated that shared goals and understanding, interests and vision were essential factors and that once stakeholders knew their roles, they were more likely to achieve common goals.

Our goal is to encourage collaborative action to reduce the burden of depression and to ensure that people suffering from depression receive more attention. -Government department official 2.

(4) **Resources.** Resources to support the policy development and implementation process were described as critical enablers. These resources included expertise and financial support from the government. Participants also noted that explicit budget allocations to support activities contributed.

The Ministry of Health is very supportive regarding resources and expertise, so we work well together. -Volunteer organisations 1.

(5) **Application of digital technology.** Using digital technologies such as artificial intelligence and big data is essential for implementing depression prevention and treatment policies. The participants said that digital technologies provide innovative treatment methods and paradigms for the “early diagnosis and treatment” of mental abnormalities.

In reality, many people avoid treatment and suffer alone because of “stigma, psychological precautions, space and time constraints”. To this day, depression is still unknown to the general public. But the rise of digital depression interventions could go some way to alleviating this situation. -Psychologist 1.

The Digital Depression Intervention Programme breaks through time and space constraints and becomes a private “psychotherapist” for the user 24 h a day, seven days a week, through one-to-one support. -Researchers 1.

## Barriers to the development and implementation of depression prevention and treatment policies

Study participants described many challenges faced during the development and implementation of depression prevention and treatment policies. Lack of awareness is a crucial barrier to the depression policy process.

1. **Lack of awareness and depression literacy.** Although depression has received increasing attention, there is still a lack of public awareness of the clinical presentation and treatment of depression. There is a significant gap in the public’s awareness and understanding of depression.

Proper awareness is only the first step; depression prevention and treatment still have a long way to go in medical and scientific fields. -Health department official 2.

Firstly, the public still lacks a comprehensive understanding of the clinical manifestations of depression. Most people do not realise that unexplained somatic symptoms may also be a clinical manifestation of depression. The lack of essential awareness of the clinical manifestations of depression prevents the public from seeking timely medical attention and early intervention when they or their family members are experiencing depressive symptoms.

Most older people are not particularly well aware of this. They may be more concerned about their sleep, their pain and their inability to eat on their own, so they make repeated visits to general hospitals. Older people suffering from depression can easily be overlooked and may attribute various symptoms to old age, preventing early intervention and treatment. -Mental health facility doctor 1.

Because of the ignorance of this illness, we give very little support to children. If people knew more, supported their child when they noticed signs of relapse and took him to a doctor, he would most likely be cured. -Family members of adolescents with depression 1.

Secondly, some respondents still have misconceptions about depression treatment, such as the belief that depression can be treated through ideological and educational work, and some exaggerate the role of medication. The low awareness of depression prevention and treatment methods may be one of the reasons for the low treatment rate of depression.

Many people confuse depression with low mood, believing that it is not an illness but a psychological problem caused by a lack of motivation and will, and therefore think that people with depression do not need professional treatment, predominantly medication, and others even believe that true depression is incurable. -Psychiatrist 2.

(2) **Lack of resources.** One of the obstacles to implementing the Depression Prevention and Control Policy is the lack of human and financial resources. The lack of human resources is cited as the reason for the slow progress of the policy. Compared to the enormous mental health needs, China has a massive shortage of doctors specialising in psychiatry. There are about 40,000 psychiatrists in China, and more than 60,000 psychiatrists are needed if the whole society’s needs are met. In the central and western regions and rural areas, there is a severe shortage of mental health workers with good training.

The complex pathogenesis of depression requires specialist diagnosis and recognition by psychiatrists. The low recognition rate of depression by non-psychiatric practitioners and the uneven quality of free psychological services on campuses and in the community make it challenging to identify some depressed patients. This phenomenon leads to misdiagnosis, mismanagement, and loss of time and access to early intervention and symptomatic treatment, exacerbated by the lack of mental health workers. -Ministry of Health official 2.

In many primary hospitals, there is a chronic shortage of psychiatrists and even fewer doctors specialising in diagnosing and treating adolescent mental illness. The related treatment capacity needs to be improved. -Psychiatrist 2.

Another issue is financial resources. In China, treatment for mental illness is expensive, time-consuming and has variable results.

Although some cities have included depression as an outpatient chronic illness in their health insurance, unlike the relatively manageable expenditure on medication, psychological treatment is often a much more significant expense for depressed patients. On the other hand, the cost of psychological treatment is hardly a simple solution through the idea of inclusion in health insurance. In the absence of health insurance assistance, it is common for patients to lose access to treatment or discontinue treatment for financial reasons. -Ministry of Health official 2.

In severe cases of depression, patients may even lose all functions completely …Comprehensive treatment, including medication, psychotherapy and rehabilitation training for cognitive functions, is also particularly costly and, together with the burden of expenditure on indirect care, adds to the financial burden of patients. -Psychiatrist 2.

(3) **Stigmatisation and social discrimination.** Widespread social Stigma and societal discrimination against mental disorders, including depressive disorders, prevent people with depressive disorders from accessing professional care and treatment.

It is difficult for people with depression to access effective psychosocial treatment and appropriate medical therapy. The high Stigma attached to depression prevents many people from getting their needed help. -Psychologist 2.

I was interpreted as lazy, pretentious and unmotivated by those around me. After being diagnosed with depression, my mother was distraught and felt like the sky was falling. When my parents didn’t understand, I thought of self-harm, and it took almost everything I had to get my family to accept that I was ill. -People with depression 1.

The discrimination and prejudice people face with mental disorders are so severe that many patients are afraid to admit, “I go to a psychiatric hospital”. How far are we from the point where most people with depressive disorders are being adequately treated? -Mental health expert 1.

There is a “stigma” on patients and their families. Many patients only seek medical help when they are bored with school, have reduced social functioning, or even take six months or even a year off school, by which time their depression has progressed to a more severe state. Patients are better treated if screening for the illness is done at an early stage. The sad thing is that some parents cannot accept that their child has depression even after seeing the diagnosis. -Education Department official 1.

(4) **Lack of united action.** The lack of united action by society is also a significant impediment to the prevention and treatment of depression.

The health system cannot solve the prevention, diagnosis and treatment of depression alone. Measures at the societal level should also be established in the fight against depression. It is not only the medical and psychological counselling systems that should be installed and relied upon but also a third system - the social support system. The latter is a more comprehensive and wide-ranging system that can play a role of “short-term diagnosis and treatment, long-term growth and companionship” to make up for the shortcomings of the existing medical and counselling systems. -Psychologist 2.

Hospitals, psychological, educational, and charity organisations work separately, without a systematic organisation or platform. Restoring social functioning to depressed patients requires effective medication, professional psychological counselling, and long-term systemic support from society. The government, medical institutions and the community must work together to help patients get out of ‘depression’ and holistically return to society. -A volunteer organisation 1.

Strategies that act together across society to reduce adverse experiences (including neglect and trauma) during childhood and throughout life are needed to reduce the incidence of depression. -Psychologist 2.

## Discussion

This study provides insights into the key enablers and barriers encountered in developing and implementing depression prevention and treatment policies in China. Facilitators of policy development and implementation include (1) political commitment and strong leadership, (2) coordination mechanisms, (3) stakeholder enthusiasm and commitment, (4) resources, and (5) the use of digital technologies. The main barriers leading to delays in policy development and implementation include (1) insufficient awareness and lack of depression literacy, (2) lack of resources and (3) stigma and social discrimination (4) lack of united action. These findings are consistent with the global literature on policy development and implementation for depression prevention and treatment.

## Political commitment and strong leadership

For depression prevention and control programmes to be successful, governments need to take all actions and commit to long-term cross-sectoral solutions to achieve population-wide coverage of these interventions [4] successfully. The experience of HIV/AIDS planning has shown that high-level political leadership is critical to the action of multisectoral coordination mechanisms. For China, the importance, attention and supervision of the Party and the government for national mental health efforts are more conducive to developing and implementing depression prevention and treatment policies.

## Coordination mechanisms

A mechanism to define roles and responsibilities must be established to ensure the smooth coordination of depression prevention and treatment programmes [4]. A successful coordination mechanism recognises the need to assign duties and holds participating sectors and stakeholders accountable in a consultative manner [4]. This situation will speed up the policy process and reduce unnecessary delays that often demotivate actors eager to see the policy implemented. The Ministry of Health in China has been leading in coordinating meetings and providing technical guidance for policy discussions, significantly contributing to programme implementation and enforcement. In addition, the policies and interventions adopted across sectors and the health sector have been significant in advancing the implementation of depression prevention and treatment programmes.

## Stakeholder enthusiasm and commitment

Efforts to promote depression prevention and treatment policies involve committed individuals passionate about depression prevention and treatment even in the face of significant disincentives [15]. Health professionals join forces with other stakeholders in government, civil society and communities, including people who experience depression and their families, clinical and public health practitioners, researchers and policymakers, to work together to support such policies and actively participate in the design and implementation of services, procedures and research to reduce the burden caused by depression [16].

## Resources

Financial and human resources have been identified as facilitators and barriers to depression prevention and treatment. Lack of funding has hindered progress in implementation in other countries [17–19]. The results show that the burden of depression remains high even among those experiencing minimal symptoms [20]. Although some cities and regions in China have included depression as an outpatient chronic illness in their medical insurance, patients are overwhelmingly reimbursed for medication and tests. However, unlike the relatively manageable expenditure on medication, psychological treatment is often a much more significant expense for depressed patients. And the lack of psychological treatment has led to poor policy implementation.

## The use of digital technologies

The use of digital technology to enhance treatment and care is an important new frontier in health care and is catalytic to the implementation of depression prevention and treatment programmes [21–23]. Digital strategies can extend the reach of other clinical interventions to underserved populations [24]. Digital technologies also offer new possibilities for screening, diagnosis, assessment and monitoring patients with depression. Computerised adaptive testing allows for more efficient, flexible and accurate administration of individually reported outcome measures [25–27]. The expansion of digital technology, which has become the standard delivery platform for mental health care during the New Coronary Pneumonia pandemic, has been demonstrated in various contexts.

## Insufficient awareness and lack of depression literacy

It is well known that in many countries, inadequate public awareness and lack of knowledge about depression can hinder the development and implementation of depression prevention and treatment policies, as has been well documented [17, 18, 28, 29]. Knowledge about strategies to obtain and maintain good mental health and depression and its treatment is known as depression literacy [30]. Knowledge of depression may be an essential determinant of help-seeking across age groups. Interventions that promote health literacy can increase service utilisation for depressive disorders [31]. Awareness-raising campaigns have been carried out in many countries, and a review in 2009 found that awareness and knowledge of depression had increased and attitudes towards people with mental illness had improved [32].

## Stigma and social discrimination

Stigma and social discrimination can also hinder the implementation of depression prevention and treatment programmes, which is consistent with the results of existing studies [33–36]. Attitudinal barriers in initiation and adherence to the treatment are shared across different countries world [37]. Stigma and social discrimination significantly hinder professional help-seeking for depressed patients and directly affect the environment in which they live and recover from their illnesses [38, 39]. Studies have found that stigmatisation of depression decreases the willingness and length of time the public seeks help for patients [38, 40]. Research has shown little evidence of a general decrease in public Stigma against depression in recent decades. For example, from 1996 to 2006, 33% of the general public in the United States believed that people suffering from depression were violent toward others, up from 32% in 1996[41]. Cross-sectional studies in Brazil, China and Ethiopia have shown a significant stigma attached to depression [42, 43].

## Lack of united action

The lack of united action as an impediment to the implementation of depression prevention and treatment programmes has been supported and demonstrated by research findings [4]. A united society-wide approach to depression prevention is justified and is associated with measures such as reducing alcohol consumption, overlapping with the successful actions taken in many countries to reduce the prevalence of coronary heart disease and several types of cancer [44]. An adequate response to depression requires the involvement of the whole of society and government, with joint action to reduce the risk of experiencing adversity and enhance protective factors. For example, pre-emptive action should be taken to stop the potentially devastating effects of poverty, income inequality, violence and other social inequalities on the mental health of populations [16].

## Conclusion

Strategies to reduce Stigma and increase knowledge of depression are now seen by many as part of a national and international approach to reducing the burden of depression. We need to actively create a social climate of understanding, acceptance and care for people with depression and raise awareness of the importance of mental health throughout society. Despite these challenges in developing and implementing a programme of work on specialised services for the prevention and treatment of depression, China has achieved relatively good results in developing and implementing the Exploring Specialised Services for the Prevention and Treatment of Depression programme due to strong government leadership. Therefore, implementing the policy must establish government leadership and a sound mechanism for mental health work that is led by the government and coordinated by departments. China’s experience can be an example to other countries of what can happen when political will/stakeholder commitment is solid. An adequate response to depression will require the involvement of society, with joint action to strengthen protective factors and ensure that those who need help can find it.

## Data Availability

The datasets generated and analysed during the current study are not publicly available due to the sensitive nature of narratives shared that may disclose participants’ identities but are available from the corresponding author on reasonable request.
